# Characteristics of three-dimensional acetabular morphology of patients with excellent outcome after rotational acetabular osteotomy over 20 years

**DOI:** 10.1186/s13018-021-02346-0

**Published:** 2021-03-15

**Authors:** Takeyuki Tanaka, Toru Moro, Hisatoshi Ishikura, Kazuaki Hashikura, Taizo Kaneko, Sakae Tanaka

**Affiliations:** 1 Orthopaedic Surgery, Sensory and Motor System Medicine, Surgical Sciences, Graduate School of Medicine, The University of Tokyo, 7-3-1 Hongo, Bunkyo-ku, Tokyo, 113-8655 Japan; 2 Division of Science for Joint Reconstruction, Graduate School of Medicine, The University of Tokyo, 7-3-1 Hongo Bunkyo-ku, Tokyo, 113-8655 Japan

**Keywords:** Rotational acetabular osteotomy, Long-term outcome, Acetabular morphology, Three-dimensional analysis

## Abstract

**Background:**

Rotational acetabular osteotomy (RAO) is a type of pelvic osteotomy performed to improve the acetabular bony coverage against the femoral head for patients with acetabular dysplasia. The acetabular bony coverage is ideally evaluated three-dimensionally; however, there is a paucity of published data regarding three-dimensional morphology in patients with long-term excellent outcome after RAO. The present study investigated the characteristics of three-dimensional acetabular morphology with long-term excellent outcome after RAO in comparison to patients with normal hip joints and those converted to total hip arthroplasty (THA) after RAO because of osteoarthritis (OA) progression.

**Methods:**

Anteroposterior plain radiograph and computed tomography data of 57 hip joints (17 joints with excellent outcome 20 years or more after RAO, 16 normal joints, and 20 joints converted to THA after RAO) were analyzed. The two-dimensional lateral center-edge (CE) angle from plain radiographs and acetabular anteversion, anterior acetabular sector angle, and posterior sector angle from computed tomography (CT) images were calculated.

**Results:**

Compared with patients converted to THA, all parameters in patients with long-term excellent outcome after RAO were similar to those in patients with normal hip joints, particularly in the three-dimensional analyses. The anterior bony coverage was excessive, whereas the posterior bony coverage was deficient in patients converted to THA after RAO. Anterior bony impingement and posterior instability may be the cause of OA progression after RAO.

**Conclusion:**

Caution must be taken to avoid rotating the separated fragment excessively to the anterior direction during RAO to prevent OA progression and achieve long-term excellent outcome.

## Background

**Fig. 1:**
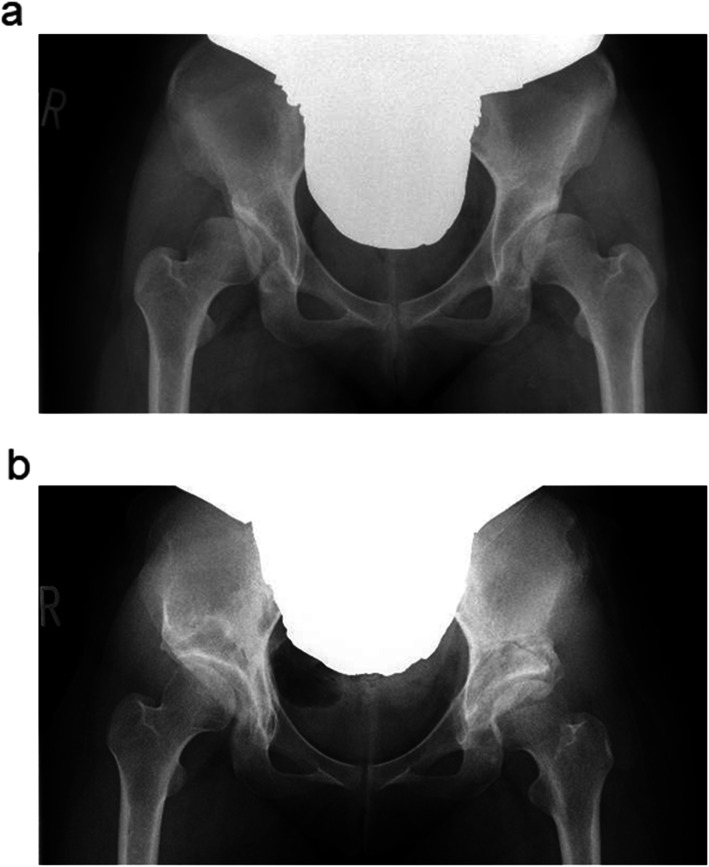
Anteroposterior radiograph of the hip joint before and after RAO. **a** Before bilateral RAO. **b** Right hip, 3 years after RAO; left hip, 3 months after RAO

Rotational acetabular osteotomy (RAO), which rotates the roundly separated acetabular bone to the lateral direction and improves the acetabular bony coverage against the femoral head, is a surgery to improve the biomechanical problems in patients with acetabular dysplasia [1, 2], including mechanical stress concentrated at the lateral edge of the acetabulum, instability of the hip joint, and increased stress to the labrum of the hip joint (Fig. 1). Favorable postoperative long-term outcomes have been reported in several studies [3–8]; however, there are some cases that require conversion to total hip arthroplasty (THA) because of the early progression of osteoarthritis (OA) after RAO [9]. These reports have focused on the two-dimensional bony coverage typified by the center-edge (CE) angle in the plain anteroposterior radiograph of the hip joint; meanwhile, the acetabular bony coverage should ideally be evaluated three-dimensionally [10, 11].

Therefore, in the present study, we hypothesized that the three-dimensional acetabular morphology differs between the patients with excellent long-term outcome after RAO and the patients who required conversion to THA after RAO. The aim of the present study was to demonstrate the three-dimensional acetabular morphology in patients with excellent long-term outcome after RAO in comparison with those in patients who required conversion to THA after RAO and patients with normal hip joint.

## Methods

### Patients

After obtaining approval from the institutional review board of our hospital, 17 patients who previously underwent RAO for 20 years or more, in whom the osteoarthritic clinical stage was at the pre- or initial stage and in whom the Japanese Orthopaedic Association hip score (JOA score) was over 90 points, were recruited (RAO group). After informed consent on the irradiation and the medical costs of computed tomography (CT) were obtained from all participants, three-dimensional investigations were performed. The JOA score consists of the following 4 categories: pain (40 points), range of motion (20 points), gait (20 points), and activities of daily living (20 points). Obtaining a JOA score of over 90 points indicates an excellent clinical condition [12]. In addition, as control groups, 20 patients who underwent THA after RAO because of an advanced clinical OA stage (THA group) and 16 patients with normal hip joint (normal group) were recruited. To evaluate the three-dimensional acetabular morphology, CT images of the pelvis and femur were obtained from all participants. In the THA group, CT examination was performed just prior to THA. The average ages in the RAO group, THA group, and normal group during CT examination were 55.0 (range 36–65), 58.2 (range 40–68), and 60.4 (range 47–69) years, respectively. There were 2 male patients in the RAO group and THA group, and 5 male patients in the normal group. The average body mass indices were 21.9 in the RAO group, 23.0 in the THA group, and 23.4 in the normal group. The average interval from RAO to CT evaluation in the RAO group was 26.7 (range 20–38) years, and the average interval from RAO to THA conversion in the THA group was 22.8 (range 8–36) years.

### Radiographic evaluation

**Fig. 2:**
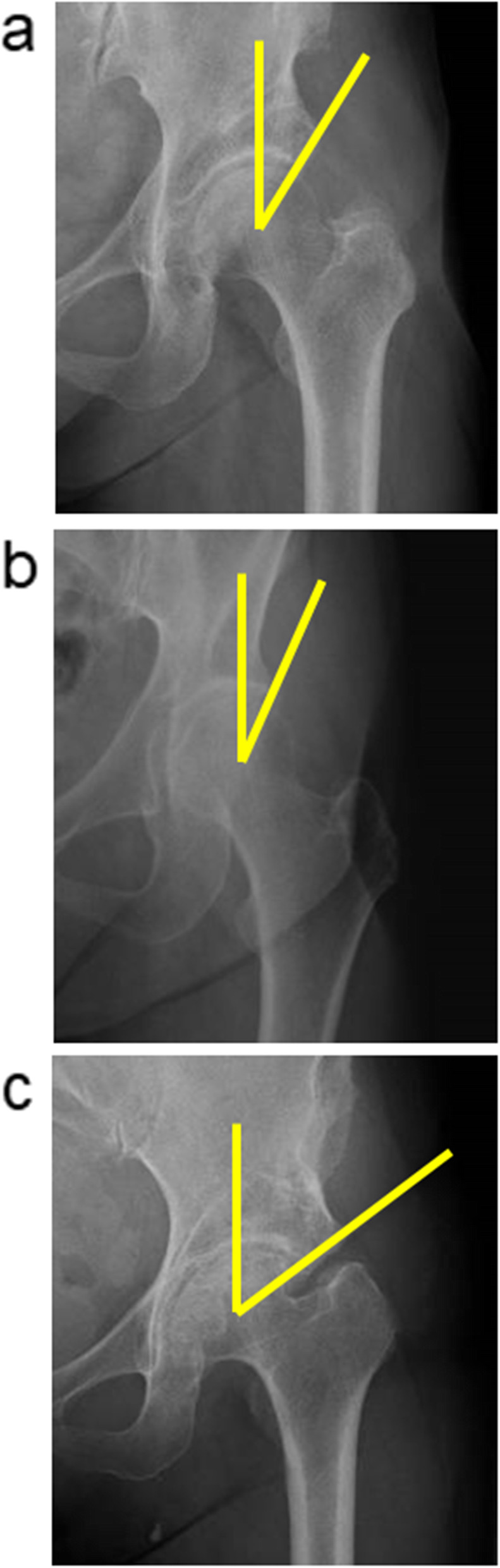
Anteroposterior radiograph of the hip joint and measurement of the lateral CE angle in each group. **a** RAO group. **b** Normal group. **c** THA group

**Fig. 3:**
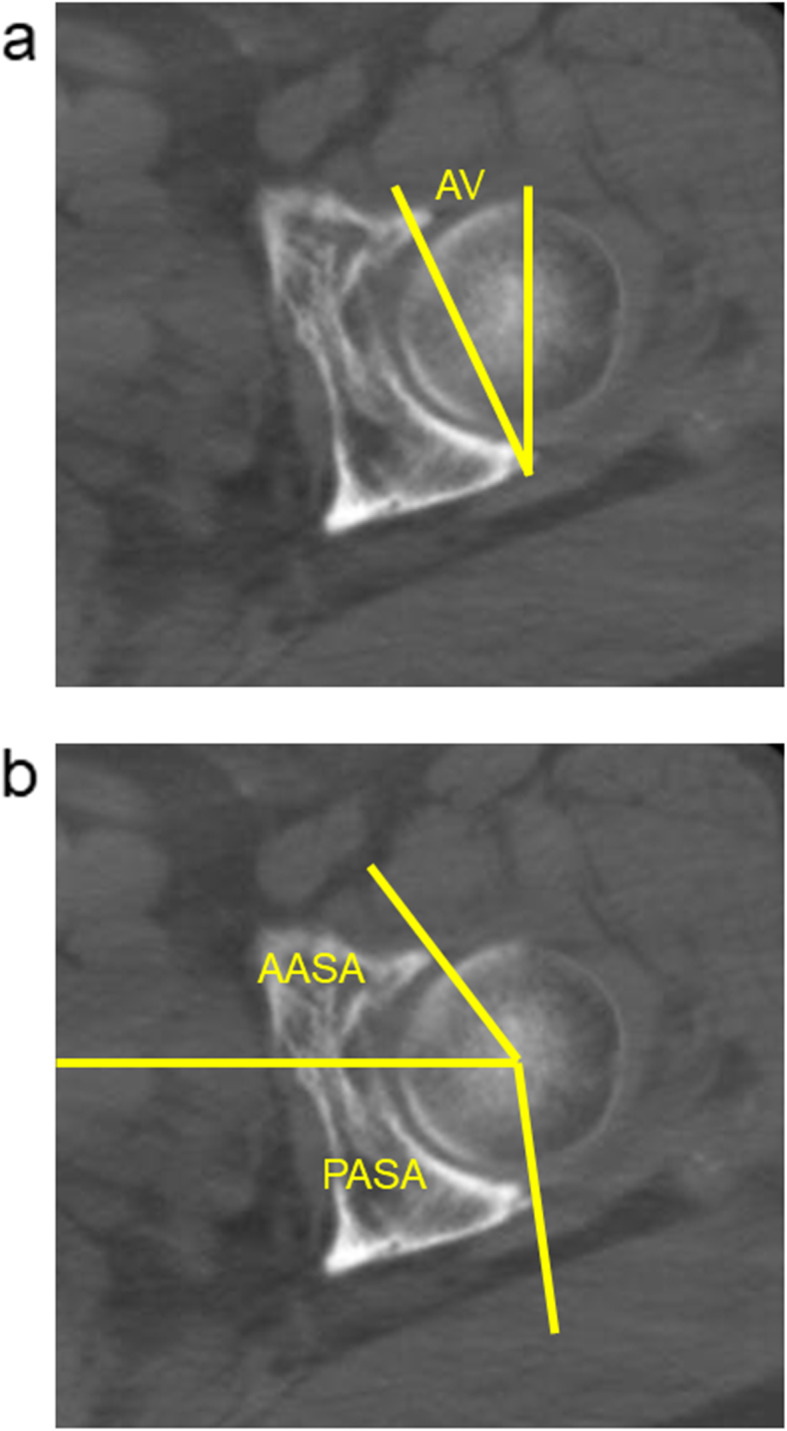
Axial slice of the CT data at the level of the femoral head center. **a** Measurement of the acetabular anteversion (AV). **b** Measurement of anterior acetabular sector angle (AASA) and posterior acetabular sector angle (PASA)

To investigate the two-dimensional acetabular bony coverage against the femoral head, the lateral CE angle in the anteroposterior plain radiograph of the hip joint was measured. In measuring the lateral CE angle, the lateral edge of the acetabulum was standardized as the most lateral point of the acetabular sourcil (Fig. 2). Furthermore, to examine the three-dimensional acetabular bony coverage against the femoral head, according to the method of Anda et al., the acetabular anteversion (AV), anterior acetabular sector angle (AASA), and posterior acetabular sector angle (PASA) were measured from the axial slice of CT data at the level of the femoral head center (Fig. 3) [13].

### Statistical analysis

Statistical analyses were conducted using Student’s *t*-test (version 2.00; Excel statistics, SSRI Co., Ltd., Tokyo, Japan). In all analyses, a *p*-value <0.05 was considered statistically significant.

## Results

### Two-dimensional evaluation

Table 1 shows the two-dimensional lateral CE angles measured by anteroposterior radiography of the bilateral hip joints in each group. The mean lateral CE angles were 40.9 ± 4.9° in the RAO group, 42.1 ± 13.8° in the THA group, and 34.3 ± 5.9° in the normal group. There were significant differences in the lateral CE angle between the normal group and other groups (*p* = 0.001 and 0.043 compared with the RAO group and THA group, respectively). There was no significant difference between the RAO group and THA group (*p* = 0.732); however, the lateral CE angle in the RAO group was more similar to that in the normal group than that in the THA group.

**Table 1:** Lateral CE angle and *p*-values between each group

	RAO group (*R*)	THA group (*T*)	*p*-value vs. *R*	Normal group (*N*)	*p*-value vs. *R*	*p*-value vs. *T*
CE angle	40.9 ± 4.9	42.1 ± 13.8	0.732	34.3 ± 5.9	0.001	0.043

Values are presented as means ± standard deviations

### Three-dimensional evaluation

Table 2 shows the AV, AASA, and PASA measured by CT examination in each group.

**Table 2:** AV, AASA, and PASA, and p-values between each group

	RAO group (*R*)	THA group (*T*)	*p*-value vs. *R*	Normal group (*N*)	*p*-value vs. *R*	*p*-value vs. *T*
AV	25.9 ± 11.7	−0.8 ± 18.5	< 0.001	20.4 ± 6.6	0.110	< 0.001
AASA	44.7 ± 19.7	77.7 ± 40.9	0.005	55.6 ± 13.3	0.033	0.071
PASA	93.1 ± 9.7	84.0 ± 11.4	0.014	100.5 ± 8.4	0.026	< 0.001

Values are presented as means ± standard deviations

The mean AV values were 25.9 ± 11.7° in the RAO group, −0.8 ± 18.5° in the THA group, and 20.4 ± 6.6° in the normal group. With regard to the AV, there were significant differences between the THA group and other groups (*p* < 0.001, compared with both groups). Moreover, in the THA group, the morphology of the acetabulum was retroverted in the supine position, which is the body positioning during CT examination, in 10 out of 20 hip joints; meanwhile, the retroverted acetabulum was present in only 1 hip joint in the RAO group, and it was not observed in the normal group. Besides, there was no significant difference between the RAO group and the normal group (*p* = 0.110).

The mean AASA values were 44.7 ± 19.7° in the RAO group, 77.7 ± 40.9° in the THA group, and 55.6 ± 13.3° in the normal group. In terms of the AASA, there were significant differences between the THA group and other groups (*p* = 0.005 and 0.033 compared with the RAO group and normal group, respectively). This result showed that the anterior aspect of the acetabulum in the THA group was more excessive than those in the RAO group and normal group. On the other hand, there was no significant difference between the RAO group and normal group (*p* = 0.071).

The mean PASA values were 93.1 ± 9.7° in the RAO group, 84.0 ± 11.4° in the THA group, and 100.5 ± 8.4° in the normal group. With regard to the PASA, there were significant differences among all groups (*p* = 0.014: RAO group vs. THA group, *p* = 0.026: RAO group vs. normal group, and *p* < 0.001: THA group vs. normal group). Meanwhile, the PASA values in the RAO group were more similar to that in the normal group than that in the THA group, and this result indicated that the posterior aspect of the acetabulum in the THA group was more deficient than that in the RAO group.

**Fig. 4:**
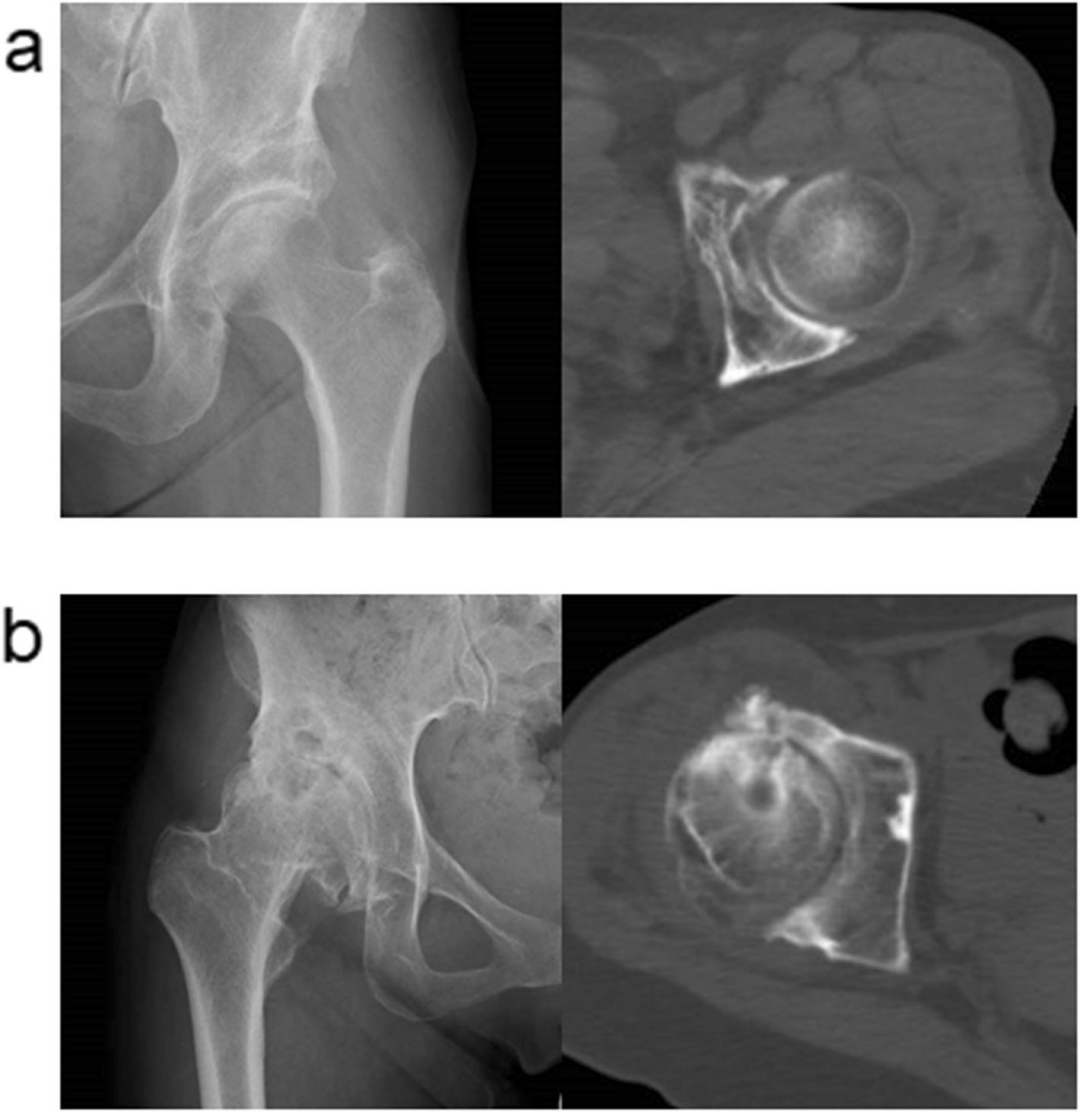
Anteroposterior radiograph and axial slice of the CT data of hip joint. **a** A hip joint 38 years after RAO in RAO group. Radiograph shows that clinical stage of osteoarthritis remained to be initial, and CT data shows three-dimensional acetabular morphology is not retroverted. **b** A hip joint 8 years after RAO in THA group. Radiograph shows that clinical stage of osteoarthritis advanced to end-stage, and CT data shows three-dimensional acetabular morphology is significantly retroverted

The results of the present study are summarized as follows: the morphology of the acetabulum in patients with excellent outcomes after RAO was more similar to that in patients with normal hip joints than that in patients converted to THA, particularly in the three-dimensional analyses. Furthermore, the acetabular morphology was retroverted in 50% of the patients converted to THA after RAO. Typical axial slices of CT data at the level of the femoral head center of RAO and THA groups are shown in Fig. 4.

## Discussion

RAO is a type of pelvic osteotomy that improves the biomechanical condition of the hip joint by rotating the separated acetabulum toward the lateral direction, which is thought to prevent OA progression in patients with acetabular dysplasia [1, 2]. Meanwhile, conversion to THA is necessary in some cases because of the early OA progression after RAO. The evaluation of the acetabular morphology should ideally be performed three-dimensionally; however, there is a paucity of published data regarding the three-dimensional acetabular morphology [10, 11], probably because of the irradiation effects or the medical costs of CT examination. Similarly, the three-dimensional acetabular morphology in other types of pelvic osteotomy was evaluated in only a few published studies [14, 15].

The present study revealed that the morphology of the acetabulum in patients with excellent outcomes after RAO was more similar to that in patients with normal hip joints than that in patients converted to THA, especially in the three-dimensional analyses. Meanwhile, the two-dimensional evaluation, typified by the lateral CE angle, was not statistically different between the RAO group and THA group. This finding indicates the limitation of the two-dimensional evaluation for the ideal RAO procedure. Moreover, the direction and extent of separated fragment rotation during RAO must be ideally approximated to the morphology of normal hip joints three-dimensionally as well as two-dimensionally. Therefore, comprehension of the three-dimensional acetabular morphology of normal hip joints is essential to achieve favorable long-term outcomes in RAO.

With respect to the morphology of the anterior and posterior aspect of the acetabulum, the present study revealed excessive anterior aspect bony coverage and deficient posterior aspect bony coverage in patients converted to THA compared to patients with long-term excellent outcomes after RAO. Several previous reports indicated anterior bony impingent between the acetabulum and femoral head, leading to progression of the osteoarthritic change, after RAO [16–18]. Meanwhile, several previous reports pointed out that a retroverted acetabulum, including postoperative or nonoperative hip, causes the progression of OA [19, 20]. In the THA group, anterior bony impingement and posterior instability may be the cause of OA progression. Considering these factors, it is important that caution must be taken to avoid rotating the separated fragment excessively to the anterior direction during RAO, to prevent the progression of osteoarthritic change after RAO in the long-term.

This study has several limitations. First, the number of the patients was small. As mentioned previously, to evaluate the three-dimensional morphology, CT data is necessary; however, because of the issue of irradiation and medical costs, the number of the participants may be limited. On the other hand, considering that published data evaluating the three-dimensional acetabular morphology in patients with long-term excellent clinical outcomes after RAO are deficient, the results of the present study provide helpful information on the ideal three-dimensional acetabular morphology in RAO. Second, the results of the present study reflected only the bony morphology of the acetabulum, and soft tissues were not evaluated. In patients with acetabular dysplasia, the condition of soft tissues, including the labrum and joint cartilage, may affect the progression of OA [21]. However, considering that the results of the present study showed the apparent differences between patients with long-term excellent results and patients converted to THA, it is definite that the evaluation of the bony morphology was important. Third, the present study only evaluated radiographic data. However, the three-dimensional CT data of patients with long-term excellent outcome are intrinsically valuable and deficient because of the problem of irradiation and medical costs, as mentioned previously. Therefore, although the number of the patients was small and the analyses were performed only radiographically, the results of the present study are meaningful for determining ideal methods for the RAO procedure. Fourth, because the radiographic examinations were performed before THA, OA progression lead the spur protrusion and might be the cause of overestimation of the parameters, in particular, the lateral CE angle and AASA in the THA group. However, in almost cases of the THA group, the extent of osteophyte formation was little or none in the anterior and lateral direction, therefore, the influence of the overestimation was thought to be considerably small. This suggested that the main cause of the OA progression after RAO was the bony morphology, as typified by acetabular retroversion or posterior instability.

## Conclusions

The acetabular morphology in patients with long-term excellent outcomes after RAO was more similar to that in patients with normal hip joints than that in patients converted to THA after RAO. Caution must be taken to avoid rotating the separated fragment excessively to the anterior direction during RAO.

## Data Availability

The datasets used/or analyzed during the current study are available from the corresponding author on reasonable request.

## Abbreviations

RAO: Rotational acetabular osteotomy
THA: Total hip arthroplasty
OA: Osteoarthritis
CE: Center-edge
CT: Computed tomography
JOA: Japanese Orthopaedic Association
AV: Acetabular anteversion
AASA: Anterior acetabular sector angle
PASA: Posterior acetabular sector angle
